# The impact of public health insurance on health care utilisation, financial protection and health status in low- and middle-income countries: A systematic review

**DOI:** 10.1371/journal.pone.0219731

**Published:** 2019-08-28

**Authors:** Darius Erlangga, Marc Suhrcke, Shehzad Ali, Karen Bloor

**Affiliations:** 1 Department of Health Sciences, University of York, York, England, United Kingdom; 2 Centre of Health Economics, University of York, York, England, United Kingdom; 3 Luxembourg Institute of Socio-economic Research (LISER), Luxembourg; 4 Department of Epidemiology and Biostatistics, Schulich School of Medicine and Dentistry, Western University, London, Ontario, Canada; University of Malta Faculty of Health Sciences, MALTA

## Abstract

**Background:**

Expanding public health insurance seeks to attain several desirable objectives, including increasing access to healthcare services, reducing the risk of catastrophic healthcare expenditures, and improving health outcomes. The extent to which these objectives are met in a real-world policy context remains an empirical question of increasing research and policy interest in recent years.

**Methods:**

We reviewed systematically empirical studies published from July 2010 to September 2016 using Medline, Embase, Econlit, CINAHL Plus via EBSCO, and Web of Science and grey literature databases. No language restrictions were applied. Our focus was on both randomised and observational studies, particularly those including explicitly attempts to tackle selection bias in estimating the treatment effect of health insurance. The main outcomes are: (1) utilisation of health services, (2) financial protection for the target population, and (3) changes in health status.

**Findings:**

8755 abstracts and 118 full-text articles were assessed. Sixty-eight studies met the inclusion criteria including six randomised studies, reflecting a substantial increase in the quantity and quality of research output compared to the time period before 2010. Overall, health insurance schemes in low- and middle-income countries (LMICs) have been found to improve access to health care as measured by increased utilisation of health care facilities (32 out of 40 studies). There also appeared to be a favourable effect on financial protection (26 out of 46 studies), although several studies indicated otherwise. There is moderate evidence that health insurance schemes improve the health of the insured (9 out of 12 studies).

**Interpretation:**

Increased health insurance coverage generally appears to increase access to health care facilities, improve financial protection and improve health status, although findings are not totally consistent. Understanding the drivers of differences in the outcomes of insurance reforms is critical to inform future implementations of publicly funded health insurance to achieve the broader goal of universal health coverage.

## Introduction

In recent decades, achieving universal health coverage (UHC) has been a major health policy focus globally.[1–3] UHC entitles all people to access healthcare services through publicly organised risk pooling,[4] safeguarding against the risk of catastrophic healthcare expenditures.[5] Low- and middle-income countries (LMICs) face particular challenges in achieving UHC due to particularly limited public resources for health care, inefficient allocation, over-reliance on out-of-pocket payments, and often large population size.[5] As a result, access to health care and the burden of financial cost in LMICs tends to be worse for the poor, often resulting in forgone care.[6–8]

Introducing and increasing the coverage of publicly organised and financed health insurance is widely seen as the most promising way of achieving UHC,[9,10] since private insurance is mostly unaffordable for the poor.[11] Historically, social health insurance, tax-based insurance, or a mix of the two have been the dominant health insurance models amongst high income countries and some LMICs, including Brazil, Colombia, Costa Rica, Mexico, and Thailand.[12] This is partly influenced by the size of the formal sector economy from which taxes and payroll contributions can be collected. In recent decades, community-based health insurance (CBHI) or “mutual health organizations” have become increasingly popular among LMICs, particularly in Sub-Saharan Africa (e.g. Burkina Faso,[13] Senegal[14] and Rwanda[15]) as well as Asia (e.g. China[16] and India[17]). CBHI has emerged as an alternative health financing strategy, particularly in cases where the public sector has failed to provide adequate access to health care.[18]

We searched for existing systematic reviews on health insurance in the Cochrane Database for Systematic Reviews, Medline, Embase, and Econlit. Search terms “health insurance”, “low-middle income countries”, and “utilisation” were used alongside methodological search strategy to locate reviews. Seven systematic reviews were identified of varying levels of quality, [19–26] with Acharya et al.[27] being the most comprehensive. The majority of existing reviews has suggested that publicly-funded health insurance has typically shown a positive impact on access to care, while the picture for financial protection was mixed, and evidence of the impact on health status was very sparse.

This study reviews systematically the recent fast-growing evidence on the impact of health insurance on health care utilisation, financial protection and health status in LMICs. Since the publication of Acharya et al. (which conducted literature searches in July 2010), the empirical evidence on the impact of health insurance has expanded significantly in terms of quantity and quality, with growing use of sophisticated techniques to account for statistical challenges[28] (particularly insurance selection bias). This study makes an important contribution towards our understanding of the impact of health insurance in LMICs, taking particular care in appraising the quality of studies. We recognise the heterogeneity of insurance schemes implemented in LMICs and therefore do not attempt to generalise findings, but we aim to explore the pattern emerging from various studies and to extract common factors that may affect the effectiveness of health insurance, that should be the focus of future policy and research. Furthermore, we explore evidence of moral hazard in insurance membership, an aspect that was not addressed in the Acharya et al review.[27]

## Methods

This review was planned, conducted, and reported in adherence with PRISMA standards of quality for reporting systematic reviews.[29]

### Participants

Studies focusing on LMICs are included, as measured by per capita gross national income (GNI) estimated using the World Bank Atlas method per July 2016.[30]

### Intervention

Classification of health insurance can be complicated due to the many characteristics defining its structure, including the mode of participation (compulsory or voluntary), benefit entitlement, level of membership (individual or household), methods for raising funds (taxes, flat premium, or income-based premium) and the mechanism and extent of risk pooling [31]. For the purpose of this review, we included all health insurance schemes organised by government, comprising social health insurance and tax-based health insurance. Private health insurance was excluded from our review, but we recognise the presence of community-based health insurance (CBHI) in many LMICs, especially in Africa and Asia [18]. We also therefore included CBHI if it was scaled up nationally or was actively promoted by national government. Primary studies that included both public and private health insurance were also considered for inclusion if a clear distinction between the two was made in the primary paper. Studies examining other types of financial incentives to increase the demand for healthcare services, such as voucher schemes or cash transfers, were excluded.

### Control group

In order to provide robust evidence on the effect on insurance, it is necessary to compare an insured group with an appropriate control group. In this review, we selected studies that used an uninsured population as the control group. Multiple comparison groups were allowed, but an uninsured group had to be one of them.

### Outcome measures

We focus on three main outcomes:

- Utilisation of health care facilities or services (e.g. immunisation coverage, number of visits, rates of hospitalisation).
- Financial protection, as measured by changes in out-of-pocket (OOP) health expenditure at household or individual level, and also catastrophic health expenditure or impoverishment from medical expenses.
- Health status, as measured by morbidity and mortality rates, indicators of risk factors (e.g. nutritional status), and self-reported health status.

The scope of this review is not restricted to any level of healthcare delivery (i.e. primary or secondary care). All types of health services were considered in this review.

### Types of studies

The review includes randomized controlled trials, quasi-experimental studies (or “natural experiments”[32]), and observational studies that account for selection bias due to insurance endogeneity (i.e. bias caused by insurance decisions that are correlated with the expected level of utilisation and/or OOP expenditure). Observational studies that did not take account of selection bias were excluded.

### Databases and search terms

A search for relevant articles was conducted on 6 September 2016 using peer-reviewed databases (Medline, Embase, Econlit, CINAHL Plus via EBSCO and Web of Science) and grey literatures (WHO, World Bank, and PAHO). Our search was restricted to studies published since July 2010, immediately after the period covered by the earlier Acharya et al. (2012) review. No language restrictions were applied. Full details of our search strategy are available in the supporting information (S1 Table).

### Screening and data extraction

Two independent reviewers (DE and MS) screened all titles and abstracts of the initially identified studies to determine whether they satisfied the inclusion criteria. Any disagreement was resolved through mutual consensus. Full texts were retrieved for the studies that met the inclusion criteria. A data collection form was used to extract the relevant information from the included studies.

### Assessment of study quality

We used the Grades of Assessment, Development and Evaluation (GRADE) system checklist[33,34] which is commonly used for quality assessment in systematic reviews. However, GRADE does not rate observational studies based on whether they controlled for selection bias. Therefore, we supplemented the GRADE score with the ‘Quality of Effectiveness Estimates from Non-randomised Studies’ (QuEENS) checklist.[35]

cRandomised studies were considered to have low risk of bias. Non-randomised studies that account for selection on observable variables, such as propensity score matching (PSM), were categorised as high risk of bias unless they provided adequate assumption checks or compared the results to those from other methods, in which case they may be classed as medium risk. Non-randomised studies that account for selection on both observables and unobservables, such as regression with difference-in-differences (DiD) or Heckman sample selection models, were considered to have medium risk of bias–some of these studies were graded as high or low risk depending on sufficiency of assumption checks and comparison with results from other methods.

Heterogeneity of health insurance programmes across countries and variability in empirical methods used across studies precluded a formal meta-analysis. We therefore conducted a narrative synthesis of the literature and did not report the effect size. Throughout this review, we only considered three possible effects: positive outcome, negative outcome, or no statistically significant effect (here defined as p-value > 0.1).

## Results

### Results of the search

**Fig 1:**
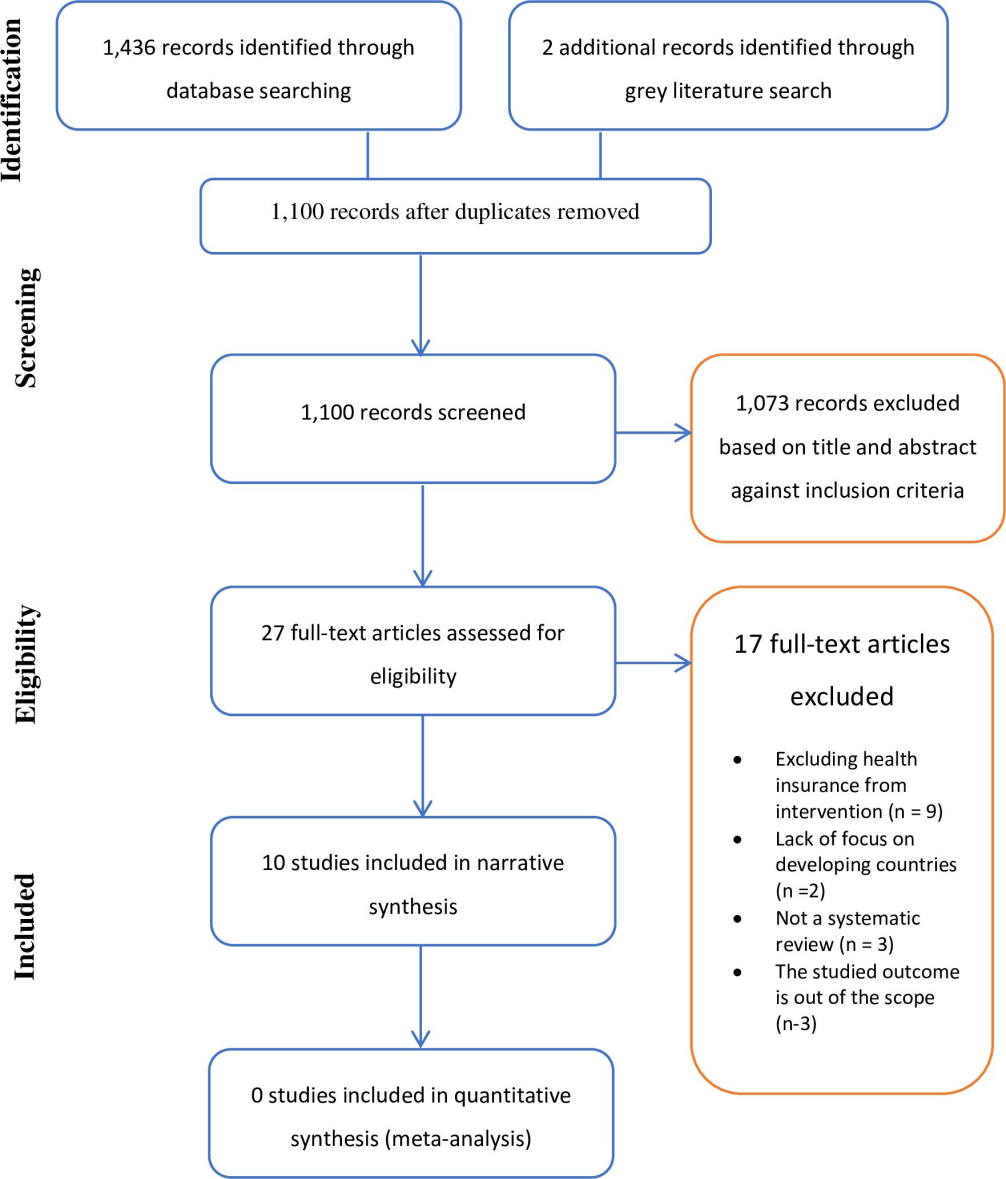
PRISMA flow diagram for included and excluded studies.

Our database search identified 8,755 studies. Five additional studies were retrieved from grey literature. After screening of titles and abstracts, 118 studies were identified as potentially relevant. After reviewing the full-texts, 68 studies were included in the systematic review (see Fig 1 for the PRISMA diagram). A full description of the included studies is presented in the supporting information (S2 Table). Of the 68 included studies, 40 studies examined the effect on utilisation, 46 studies on financial protection, and only 12 studies on health status (see Table 1).

**Table 1:** Summary of the impact of health insurance on utilisation, financial protection, and health outcomes.

QUEENS* score and GRADE	Utilisation (N = 40)				Financial protection† (N = 46)				Health status (N = 12)			
	Positive effect	No effect	Negative effect	Total	Positive effect	No effect	Negative effect	Total	Positive effect	No effect	Negative effect	Total
3 and Moderate	1	1	0	2	3	1	0	4	2	0	1	3
3 and Low	6	0	1	7	3	4	1	8	2	0	0	2
2 and Low	15	3	0	18	15	8	1	24	3	2	0	5
1 and Low	10	3	0	13	4	4	2	10	2	0	0	2
Total	32	7	1	40	25	17	4	46	9	2	1	12

* QUEENS score: 1 = high risk of bias; 2 = moderate risk; 3 = low risk; GRADE score: Low = low quality; Moderate = moderate quality; High = high quality

† Positive effect for financial protection means that health insurance decreases out-of-pocket health expenditure or reduces the event of catastrophic health expenditure

### Utilisation of health care

Table 2 collates evidence on the effects of health insurance on utilisation of healthcare services. Three main findings were observed:

1. Evidence on utilisation of curative care generally suggested a positive effect, with 30 out of 38 studies reporting a statistically significant positive effect.
2. Evidence on preventive care is less clear with 4 out of 7 studies reporting a positive effect, two studies finding a negative effect and one study reporting no effect.
3. Among the higher quality studies, i.e. those that suitably controlled for selection bias reflected by moderate or low GRADE score and low risk of bias (score = 3) on QuEENS, seven studies reported a positive relationship between insurance and utilisation. One study[36] reported no statistically significant effect, and another study found a statistically significant negative effect.[37]

**Table 2:** Summary of studies reporting utilisation of health care (N = 40), by countries and year.

Study	Year	Country/Area	Type/name of insurance*	Effect	QUEENS**	GRADE^†^
Robyn et al[13]	2012	Burkina Faso, Nouna district	CBHI	0	3	Moderate
Robyn et al[38]	2012	Burkina Faso, Nouna district	CBHI	+	1	Low
Levine, Polimeni, and Ramage[39]	2016	Cambodia	CBHI	+	3	Moderate
Babiarz et al[40]	2010	China	NCMS (Voluntary)	0	2	Low
Lu, Liu, and Shen[41]	2012	China	NCMS (Voluntary)	+	2	Low
Chen et al[42]	2014	China	URBMI (Voluntary)	+	2	Low
Hou et al[43]	2014	China	NCMS (Voluntary)	+	2	Low
Liu and Zhao[44]	2014	China	URBMI (Voluntary)	+	2	Low
Cheng et al[45]	2015	China	NCMS (Voluntary)	+	2	Low
Liao, Gilmour, and Shibuya[46]	2016	China	All public insurance	+	1	Low
Trujillo et al[47]	2010	Colombia	Voluntary and subsidised scheme	+	2	Low
Hassan et al[48]	2013	Colombia	Subsidised scheme	+	1	Low
Miller et al[49]	2013	Colombia	Subsidised scheme	+	3	Low
Hou and Chao[50]	2011	Georgia	MIP (Subsidised scheme)	+	3	Low
Zoidze et al[51]	2013	Georgia	MIP (Subsidised scheme)	0	1	Low
Gotsadze et al[52]	2015	Georgia	MIP (Subsidised scheme)	0	1	Low
Blanchet et al[53]	2012	Ghana	NHIS (Voluntary scheme)	+	1	Low
Yilma et al[54]	2012	Ghana	NHIS (Voluntary scheme)	+	3	Low
Abrokwah et al[55]	2014	Ghana	NHIS (Voluntary scheme)	+	1	Low
Brugiavini and Pace[56]	2015	Ghana	NHIS (Voluntary scheme)	+	2	Low
Fenny et al[57]	2015	Ghana	NHIS (Voluntary scheme)	+	1	Low
Sheth[37]	2014	India (Maharashtra)	CBHI	-	3	Low
Sood et al[58]	2014	India (Karnataka)	Subsidised scheme	0	2	Low
Raza et al[36]	2016	India (Uttar Pradesh and Bihar)	CBHI	0	3	Moderate
Sparrow et al[59]	2013	Indonesia	JKN (Voluntary and subsidised)	+	2	Low
Alkenrack and Lindelow[60]	2015	Lao PDR	CBHI	+	2	Low
Rivera-Hernandez et al[61]	2016	Mexico	Seguro Popular (Voluntary scheme)	0	2	Low
Bernal et al[62]	2014	Peru	SIS (Subsidised scheme)	+	3	Low
Dhillon et al[63]	2012	Rwanda (Mayange, Mwogo and Mareba)	CBHI	+	1	Low
Lu et al[64]	2012	Rwanda (all rural area)	CBHI	+	2	Low
Panpiemras et al[65]	2011	Thailand	UCS (subsidised scheme)	+	1	Low
Ghislandi, Manachotphong, and Perego[66]	2015	Thailand	UCS (subsidised scheme)	+	2	Low
Limwattananon et al[67]	2015	Thailand	UCS (subsidised scheme)	+	2	Low
Makhloufi et al[68]	2015	Tunisia	MHI (Contributory) and MAS (Subsidised)	+	1	Low
Nguyen[69]	2012	Vietnam	All public insurance	+	3	Low
Nguyen and Wang[70]	2013	Vietnam	Subsidised scheme for children	+	2	Low
Guindon[71]	2014	Vietnam	Subsidised scheme	+	2	Low
Nguyen[72]	2014	Vietnam	Contributory (compulsory and voluntary) scheme	+	1	Low
Palmer et al[73]	2015	Vietnam	Subsidised scheme for children	+	3	Low
Nguyen[74]	2016	Vietnam	Voluntary and subsidised scheme (children)	+	2	Low

* SHI = Social Health Insurance; CBHI = Community-based Health Insurance

** Queens score: 1 = high risk of bias; 2 = moderate risk; 3 = low risk

† Grade score: Low = low quality; Moderate = moderate quality; High = high quality

### Financial protection

Overall, evidence on the impact of health insurance on financial protection is less clear than that for utilisation (see Table 3). 34 of the 46 studies reported the impact of health insurance on the level of out-of-pocket health expenditure. Among those 34 studies, 17 found a positive effect (i.e. a reduction in out-of-pocket expenditure), 15 studies found no statistically significant effect, and two studies–from Indonesia[59] and Peru[62]–reported a negative effect (i.e. an increase in out-of-pocket expenditure).

**Table 3:** Summary of studies reporting financial protection outcome (N = 46).

Study	Year	Country	Insurance*	Cost sharing	Effect	QUEENS**	GRADE^†^
Parmar et al[81]	2012	Burkina Faso, Nouna district	CBHI	Yes	+	2	Low
Fink et al[75]	2013	Burkina Faso, Nouna district	CBHI	Yes	+	3	Moderate
Levine, Polimeni, and Ramage[39]	2016	Cambodia	CBHI	No	+	3	Moderate
Babiarz et al[40]	2010	China	NCMS (Voluntary)	Yes	+	2	Low
Lu, Liu, and Shen[41]	2012	China	NCMS (Voluntary)	Yes	0	2	Low
Cheung and Padieu[80]	2013	China	NCMS (Voluntary)	Yes	+	2	Low
Jing et al[82]	2013	China	NCMS (Voluntary)	Yes	0	1	Low
Bai and Wu[79]	2014	China	NCMS (Voluntary)	Yes	+	3	Low
Hou et al[43]	2014	China	NCMS (Voluntary)	Yes	0	2	Low
Liu and Zhao[44]	2014	China	URBMI (Voluntary)	Yes	0	2	Low
Liu, Wu, and Liu[83]	2014	China	All public insurance	Yes	0	1	Low
Yuan et al[84]	2014	China	All public insurance	Yes	0	2	Low
Atella, Brugiavini, and Pace[85]	2015	China	All public insurance	Yes	+	1	Low
Cheng et al[86]	2015	China	NCMS (Voluntary)	Yes	0	2	Low
Jung and Streeter[87]	2015	China	All public insurance	Yes	+	2	Low
Yang and Wu[88]	2015	China	NCMS	Yes	0	2	Low
Camacho and Conover[89]	2013	Colombia	Subsidised scheme	No	0	3	Low
Miller et al[49]	2013	Colombia	Subsidised scheme	No	0	3	Low
Yilma et al[90]	2015	Ethiopia	CBHI	Yes	+	1	Low
Zoidze et al[51]	2013	Georgia	MIP (Subsidised scheme)	Yes	-	1	Low
Gotsadze et al[52]	2015	Georgia	MIP (Subsidised scheme)	Yes	0	1	Low
Abrokwah et al[55]	2014	Ghana	NHIS (Voluntary scheme)	Yes	+	1	Low
Brugiavini and Pace[56]	2015	Ghana	NHIS (Voluntary scheme)	Yes	0	2	Low
Aryeetey et al[78]	2016	Ghana	NHIS (Voluntary scheme)	Yes	+	2	Low
Fan et al[77]	2012	India (Andrha Pradesh)	Subsidised scheme	No	+	3	Low
Sheth[37]	2014	India (Maharashtra)	CBHI	Yes	+	3	Low
Sood et al[58]	2014	India (Karnataka)	Subsidised scheme	No	+	2	Low
Raza et al[36]	2016	India (Uttar Pradesh and Bihar)	CBHI	Yes	0	3	Moderate
Aji et al[91]	2013	Indonesia	Askeskin (Subsidised scheme)	No	+	2	Low
Sparrow et al[59]	2013	Indonesia	Askeskin (Subsidised scheme)	No	-	2	Low
Alkenbrack and Lindelow[60]	2015	Lao	CBHI	Yes	0	2	Low
Galarraga et al[92]	2010	Mexico	Seguro Popular (Voluntary scheme)	Yes	+	2	Low
Sosa-Rubi, Salinas-Rodriguez, and Galarraga[93]	2011	Mexico	Seguro Popular (Voluntary scheme)	Yes	+	2	Low
Wirtz et al[94]	2012	Mexico	Seguro Popular (Voluntary scheme)	Yes	+	2	Low
Avilla-Burgos et al[95]	2013	Mexico	Seguro Popular (Voluntary scheme)	Yes	+	1	Low
Grogger et al[76]	2015	Mexico	All public insurance	Yes	+	3	Moderate
Bernal et al[62]	2014	Peru	SIS (Subsidised scheme)	No	-	3	Low
Lu et al[64]	2012	Rwanda	CBHI	Yes	+	2	Low
Koch and Alaba[96]	2010	South Africa	Contributory scheme	yes	-	1	Low
Limwattananon et al[67]	2015	Thailand	UCS (subsidised scheme)	No	+	2	Low
Makhloufi et al[68]	2015	Tunisia	MHI (Contributory) and MAS (Subsidised)	Yes	0	1	Low
Sepehri et al[97]	2011	Vietnam	Contributory, voluntary, and subsidised schemes	No	+	2	Low
Nguyen[69]	2012	Vietnam	All public insurance	Yes	0	3	Low
Nguyen and Wang[70]	2013	Vietnam	Subsidised scheme for children	No	+	2	Low
Palmer et al[73]	2015	Vietnam	Subsidised scheme for children	No	0	3	Low
Nguyen[74]	2016	Vietnam	Voluntary and subsidised scheme (children)	No	+	2	Low

* SHI = Social Health Insurance; CBHI = Community-based Health Insurance

** Queens score: 1 = high risk; 2 = moderate risk; 3 = low risk

† Grade score: Low = low quality; Moderate = moderate quality; High = high quality

Another financial protection measure is the probability of incurring catastrophic health expenditure defined as OOP exceeding a certain threshold percentage of total expenditure or income. Of the 14 studies reporting this measure, nine reported reduction in the risk of catastrophic expenditure, three found no statistically significant difference, and two found a negative effect of health insurance. Only four studies reported sensitivity analysis varying changes in the threshold level,[59,62,75,76] though this did not materially affect the findings.

Two studies used a different measure of financial protection, the probability of impoverishment due to catastrophic health expenditure, reporting conflicting findings.[77,78] Finally, four studies evaluated the effect on financial protection by assessing the impact of insurance on non-healthcare consumption or saving behaviour, such as non-medical related consumption[79], probability of financing medical bills via asset sales or borrowing[40], and household saving[80]. No clear pattern can be observed from those four studies.

### Health status

Improving health is one of the main objectives of health insurance, yet very few studies thus far have attempted to evaluate health outcomes. We identified 12 studies, with considerable variation in the precise health measure considered (see Table 4). There was some evidence of positive impact on health status: nine studies found a positive effect, one study reported a negative effect, and two studies reported no effect.

**Table 4:** Summary of studies reporting health status (N = 12).

Study	Year	Country	Insurance*	Effect	QUEENS**	GRADE†	Chosen outcomes
Fink et al[75]	2013	Burkina Faso, Nouna district	CBHI	-	3	Moderate	Child and adult mortality
Levine, Polimeni, and Ramage[39]	2016	Cambodia	CBHI	+	3	Moderate	Health index
Chen and Jin[98]	2012	China	NCMS (Voluntary)	0	2	Low	Child and maternal mortality
Cheng et al[45]	2015	China	NCMS (Voluntary)	0	2	Low	Adult mortality
Peng and Conley[99]	2015	China	NCMS (Voluntary)	+	2	Low	Malnutrition and food consumption
Camacho and Conover[89]	2013	Colombia	Subsidised scheme	+	3	Low	Low birth weight and newborn health status
Miller et al[49]	2013	Colombia	Subsidised scheme	+	3	Low	Acute illness
Sood et al[58]	2014	India (Karnataka)	Subsidised scheme	+	2	Low	Adult mortality
Pfutze[100]	2014	Mexico	Seguro Popular (Voluntary scheme)	+	2	Low	Child mortality
Pfutze[101]	2015	Mexico	Seguro Popular (Voluntary scheme)	+	1	Low	Miscarriages prevalence
Hendriks et al[102]	2014	Nigeria	CBHI	+	1	Low	Blood pressure
Quimbo et al[103]	2011	Philippines	PhilHealth (Voluntary scheme)	+	3	Moderate	CRP-positive level and wasting

* SHI = Social Health Insurance; CBHI = Community-based Health Insurance

** Queens score: 1 = high risk; 2 = moderate risk; 3 = low risk

† Grade score: Low = low quality; Moderate = moderate quality; High = high quality

### Type of insurance and countries

Considering the heterogeneity of insurance schemes among different countries, we attempted to explore the aggregate results by the type of insurance scheme and by country. Table 5 provides a summary of results classified by three type of insurance scheme: community-based health insurance, voluntary health insurance (non-CBHI), and compulsory health insurance. This division is based on the mode of participation (compulsory vs voluntary), which may affect the presence of adverse selection and moral hazard. Premiums are typically community-rated in CBHI, risk-rated in voluntary schemes and income-rated in compulsory schemes.

**Table 5:** Summary of results based on type of insurance, only 18 studies with high quality and low risk of bias.

Country and type of insurance scheme	Summary
Community-based health insurance/CBHI (N = 5 studies)	Overall: positive effect on utilisation but two studies from India shows no positive effect. Positive effect on financial protection but not in India. Contentious effect on health status
1. Burkina Faso	Positive effect on utilisation [38], reduced catastrophic health expenditure events, but negative effect on elderly’s health, ≥ 65 years old.[75] No effect among adults < 65 years old and children.[75]
2. Cambodia	Positive effect on utilisation, reduced OOP health expenditure, but no effect on health status [39]
3. India	Reduced consumption of health care but the author argued it could be that the enrolees were becoming healthier reducing their need to seek care.[37] Another paper shows no effect on utilisation and OOP health expenditure [36]
Voluntary health insurance, non-CBHI (N = 7 studies)	Overall: positive effect on utilisation. Inconclusive on financial protection, but there is an indication it is affected by rurality and proximity to adequately-staffed health facilities. Positive effect on specific health status, but not mortality rate.
1. Ghana	Positive effect on utilisation [54]
2. Vietnam	Positive effect on utilisation but no effect on OOP health expenditure [69]
3. Mexico	Reduced health expenditure among enrolees living in rural area and proximate to large health facilities. Similar effect among the enrolees living in urban area [76]
4. Philippines	Positive effect on health status measured by wasting and C-reactive protein level [103]
5. China	Positive effect on utilisation among the elderly enrolees (>65 years old) [45]; no effect on OOP health expenditure [45] but reduced non-medical consumption among the enrolees[79]; No effect on mortality rate [45,98]
Compulsory health insurance, non-CBHI (N = 6 studies)	Overall: positive effect on utilisation. Inconclusive evidence on financial protection, positive effect on specific health status.
1. India	Reduced OOP health expenditure for inpatient care [77]
2. Vietnam	Positive effect on utilisation but not on OOP health expenditure [73]
3. Georgia	Positive effect on utilisation [50]
4. Peru	Positive effect on utilisation of preventive and curative care, but negative effect on OOP health expenditure [62]
5. Colombia	Positive effect on utilisation and reduced OOP health expenditure [49]Positive effect on newborn health measured by incidence of low birth weight and several indicators of preterm baby [89]

In principle, CBHI is also considered a voluntary scheme, but we separated it to explore whether the larger size of pooling from non-CBHI schemes may affect the outcomes. Social health insurance is theoretically a mandatory scheme that requires contribution from the enrolees. However, in the context of LMICs, the mandatory element is hard to enforce, and in practice the scheme adopts a voluntary enrolment. Additionally, the government may also want to subsidise the premium for poor people. Therefore, in this review SHI schemes can fall into either the voluntary health insurance (non-CBHI) or compulsory health insurance (non-CBHI), depending on the target population defined in the evaluation study. Lastly, we chose studies with high quality/low risk only to provide more robust results.

Based on the summary in Table 5, the effect on utilisation overall does not differ based on type of insurance, with most evidence suggesting an overall increase in utilisation by the insured. The two studies showing no effect or reduced consumption of care were conducted in two different areas of India, which may–somewhat tentatively–suggest a common factor unique to India’s health system that may compromise the effectiveness of health insurance in increasing utilisation.

Regarding financial protection, the evidence for both CBHI and non-CBHI voluntary health insurance is inconclusive. Furthermore, there is an indication of heterogeneity by supply side factors captured by proximity to health facilities. Evidence from studies exploring subsidised schemes suggests no effect on financial protection, even a negative effect among the insured in Peru.

Lastly, evidence for health status may be influenced by how health outcomes are measured. Studies exploring specific health status, (examples included health indexes, wasting, C-reactive protein, and low birth weight), show a positive effect, whereas studies using mortality rates tends to show no effect or even negative effects. Studies exploring CBHI scheme did not find any evidence of positive effect on health status, as measured either by mortality rate or specific health status.

## Discussion

This review synthesises the recent, burgeoning empirical literature on the impact of health insurance in LMICs. We identified a total of 68 eligible studies over a period of six years–double the amount identified by the previous review by Acharya et al. over an approximately 60-year time horizon (1950—July 2010). We used two quality assessment checklists to scrutinise the study methodology, taking more explicit account of the methodological robustness of non-experimental designs.

Programme evaluation has been of interest to many researchers for reporting on the effectiveness of a public policy to policymakers. In theory, the gold standard for a programme evaluation is the randomised control trial, in which the treatment is randomly assigned to the participants. The treatment assignment process has to be exogenous to ensure that any observed effect between the treated and control groups can only be caused by the difference in the treatment assignment. Unfortunately, this ideal scenario is often not feasible in a public policy setting. Our findings showed that only three papers between 2010 and 2016 were able to conduct a randomised study to evaluate the impact of health insurance programmes in developing countries, particularly CBHI [38,75,103]. Policymakers may believe in the value of an intervention regardless of its actual evidence base, or they may believe that the intervention is beneficial and that no one in need should be denied it. In addition, policymakers are inclined to demonstrate the effectiveness of an intervention that they want implemented in the most promising contexts, as opposed to random allocation [104].

Consequently, programme evaluators often have to deal with a non-randomised treatment assignment which may result in selection bias problems. Selection bias is defined as a spurious relationship between the treatment and the outcome of interest due to the systematic differences between the treated and the control groups [105]. In the case of health insurance, an individual who chooses to enrol in the scheme may have different characteristics to an individual who chooses not to enrol. When those important characteristics are unobservable, the analyst needs to apply more advanced techniques and, sometimes, stronger assumptions. Based on our findings, we noted several popular methods, including propensity score matching (N = 8), difference-in-difference (N = 10), fixed or random effects of panel data (N = 6), instrumental variables (N = 12) and regression discontinuity (N = 6). Those methods have varying degree of success in controlling the unobserved selection bias and analysts should explore the robustness of their findings by comparing initial findings with other methods by testing important assumptions. We noted some papers combining two common methods, such as difference-in-difference with propensity score matching (N = 10) and fixed effects with instrumental variables (N = 8), in order to obtain more robust results.

### Overall effect

Compared with the earlier review, our study has found stronger and more consistent evidence of positive effects of health insurance on health care utilisation, but less clear evidence on financial protection. Restricting the evidence base to the small subset of randomised studies, the effects on financial protection appear more consistently positive, i.e. three cluster randomised studies[39,75,76] showed a decline in OOP expenditure and one randomised study[36] found no significant effect.

Besides the impact on utilisation and financial protection, this review identified a number of good quality studies measuring the impact of health insurance on health outcomes. Twelve studies were identified (i.e. twice as many as those published before 2010), nine of which showed a beneficial health effect. This holds for the subset of papers with stronger methodology for tackling selection bias.[39,49,89,103] In cases where a health insurance programme does not have a positive effect on either utilisation, financial protection, and health status, it is particularly important to understand the underlying reasons.

### Possible explanation of heterogeneity

#### Payment system

Heterogeneity of the impact of health insurance may be explained by differences in health systems and/or health insurance programmes. Robyn et al. (2012) and Fink et al (2013) argued that the lack of significant effect of insurance in Burkina Faso may have been partially influenced by the capitation payment system. As the health workers relied heavily on user fees for their income, the change of payment system from fee-for-services to capitation may have discouraged provision of high quality services. If enrolees perceive the quality of contracted providers as bad, they might delay seeking treatment, which in turn could impact negatively on health.

Several studies from China found the utilisation of expensive treatment and higher-level health care facilities to have increased following the introduction of the insurance scheme.[41,44,45,88] A fee-for-service payment system may have incentivised providers to include more expensive treatments.[43,83,88] Recent systematic reviews suggested that payment systems might play a key role in determining the success of insurance schemes,[23,106] but this evidence is still weak, as most of the included studies were observational studies that did not control sufficiently for selection bias.

#### Uncovered essential items

Sood et al. (2014) found no statistically significant effect of community-based health insurance on utilisation in India. They argued that this could be caused by their inability to specify the medical conditions covered by the insurance, causing dilution of a potential true effect. In other countries, transportation costs[69] and treatments that were not covered by the insurance[59,60] may explain the absence of a reduction in out-of-pocket health expenditures.

#### Methodological differences

Two studies in Georgia evaluated the same programme but with different conclusions.[50,51] This discrepancy may be explained by the difference in the estimated treatment effect: one used average treatment effect (ATE), finding no effect, and another used average treatment effect on the treated (ATT), reporting a positive effect. ATE is of prime interest when policymakers are interested in scaling up the programme, whereas ATT is useful to measure the effect on people who were actually exposed to insurance.[107]

#### Duration of health insurance

We also found that the longer an insurance programme has been in place prior to the timing of the evaluation, the higher the odds of improved health outcomes. It is plausible that health insurance would not change the health status of population instantly upon implementation.[21] While there may be an appetite among policymakers to obtain favourable short term assessments, it is important to compare the impact over time, where feasible.

#### Moral hazard

Acharya et al (2012) raised an important question about the possibility of a moral hazard effect as an unintended consequence of introducing (or expanding) health insurance in LMICs. We found seven studies exploring ex-ante moral hazard by estimating the effect on preventive care. If uninsured individuals expect to be covered in the future, they may reduce the consumption of preventive care or invest less in healthy behaviours.[108,109] Current overall evidence cannot suggest a definite conclusion considering the heterogeneity in chosen outcomes. One study found that the use of a self-treated bed nets to prevent malaria declined among the insured group in Ghana[54] while two studies reported an increase in vaccination rates[62] and the number of prenatal care visits[55,62]among the insured group. Another study reported no evidence that health insurance encouraged unhealthy behaviour or reduction of preventive efforts in Thailand.[66]

Two studies from Colombia found that the insured group is more likely to increase their demand for preventive treatment.[47,49] As preventive treatment is free for all, both authors attributed this increased demand to the scheme’s capitation system, incentivising providers to promote preventive care to avoid future costly treatments.[110] Another study of a different health insurance programme in Colombia found an opposite effect.[48]

#### Study limitations

This review includes a large variety of study designs and indicators for assessing the multiple potential impacts of health insurance, making it hard to directly compare and aggregate findings. For those studies that used a control group, the use of self-selected controls in many cases creates potential bias. Studies of the effect of CBHI are often better at establishing the counterfactual by allowing the use of randomisation in a small area, whereas government schemes or social health insurance covering larger populations have limited opportunity to use randomisation. Non-randomised studies are more susceptible to confounding factors unobserved by the analysts. For a better understanding of the links between health insurance and relevant outcomes, there is also a need to go beyond quantitative evidence alone and combine the quantitative findings with qualitative insights. This is particularly important when trying to interpret some of the counterintuitive results encountered in some studies.

## Conclusion

The impact of different health insurance schemes in many countries on utilisation generally shows a positive effect. This is aligned with the supply-demand theory in whichhealth insurance decreases the price of health care services resulting in increased demand. It is difficult to draw an overall conclusion about the impact of health insurance on financial protection, most likely because of differences in health insurance programmes. The impact of health insurance on health status suggests a promising positive effect, but more studies from different countries is required.

The interest in achieving UHC via publicly funded health insurance is likely to increase even further in the coming years, and it is one of the United Nation’s Sustainable Development Goals (SDGs) for 2030[111]. As public health insurance is still being widely implemented in many LMICs, the findings from this review should be of interest to health experts and policy-makers at the national and the international level.

## Supporting information

S1 Table Search strategies. (DOCX)

S2 Table Study characteristic and reported effect from the included studies (N = 68). (DOCX)

S3 Table PRISMA 2009 checklist. (DOCX)
