# Correction: The impact of public health insurance on health care utilisation, financial protection and health status in low- and middle-income countries: A systematic review

**DOI:** 10.1371/journal.pone.0225237

**Published:** 2019-11-07

**Authors:** Darius Erlangga, Marc Suhrcke, Shehzad Ali, Karen Bloor

The authors are listed out of order. Please view the correct author order, affiliations, and citation here:

Darius Erlangga^1^, Marc Suhrcke^2,3^, Karen Bloor^1^, Shehzad Ali^1,4^

**1** Department of Health Sciences, University of York, York, England, United Kingdom, **2** Centre of Health Economics, University of York, York, England, United Kingdom, **3** Luxembourg Institute of Socio-economic Research (LISER), Luxembourg, **4** Department of Epidemiology and Biostatistics, Schulich School of Medicine and Dentistry, Western University, London, Ontario, Canada

Erlangga D, Suhrcke M, Bloor K, Ali S (2019) The impact of public health insurance on health care utilisation, financial protection and health status in low- and middle-income countries: A systematic review. PLoS ONE 14(8): e0219731. https://doi.org/10.1371/journal.pone.0219731
