# Supplementary material for: The impact of public health insurance on health care utilisation, financial protection and health status in low- and middle-income countries: A systematic review
Source: PLoS One. 2019 Aug 28;14(8):e0219731. doi: 10.1371/journal.pone.0219731 (PMC6713352; doi:10.1371/journal.pone.0219731)
Supplement: S1 Table — (DOCX) [file pone.0219731.s001.docx]

S1 Table. Search strategies

| Databases | **CINAHL Plus** |
| --- | --- |
| Host | EBSCO |
| Date of search | 8 September 2016 |
| Years covered | January 2010 to September 2016 |
| Search strategy | S17 S15 AND S16  S16 Limiters to 2010-2016  S15 S7 AND S14  S14 S8 OR S9 OR S10 OR S11 OR S12 OR S13  S13 TI (Albania or Algeria or Angola or Argentina or Azerbaijan or Belarus or “Bosnia and Herzegovina” or Botswana or Brazil or Bulgaria or China or Colombia or “Costa Rica” or Cuba or “Dominican Republic” or Ecuador or Gabon or Hungary or Iran or Iraq or Jamaica or Jordan or Kazakhstan or Lebanon or Libya or Malaysia or Mauritius or Mexico or Montenegro or Namibia or Panama or Peru or Romania or Serbia or “South Africa” or Thailand or Yugoslav or Macedonia or Tunisia or Turkey or Turkmenistan or Venezuela  S12 TI (Armenia or Bolivia or Cameroon or “Cape Verde” or Congo or “Côte d’Ivoire” or Djibouti or Egypt or “El Savador” or Georgia or Ghana or Guatemala or Guyana or Honduras or Indonesia or Lesotho or Mauritania or Moldova or Lorocco or Nicaragua or Nigeria or Pakistan or “Papua New Guinea” or Paraguay or Phillippines or “Sao Tome and Principe” or Senegal or “Sri Lanka” or Sudan or Syrian or Ukraine or Uzbekistan or Vietnam or Yemen or Zambia) S11 TI (South Sudan or Kiribati or Lao or Samoa or “Solomon Islands” or “Timor Leste” or Tuvalu or Vanuatu or Afghanistan or Bhutan)  S10 TI (Bangladesh or Benin or “Burkina Faso” or Burundi or “Central African Republic” or Chad or Comoros or Congo or Kongo or Eritrea or Ethiopia or Gambia or Guinea or “Guinea-Bissau” or Haiti or Kenya or Kyrgyz or Liberia or Madagascar or Malawi or Mali or Mozambique or Myanmar or Nepal or Niger or Rwanda or “Sierra Leone” or Somalia or Tajikistan or Tanzania or Togo or Uganda or Zimbabwe)  S9 (MM "Developing Countries")  S8 ("developing countr*" or "developing countr*" or "low income countr*" or "middle income countr*" or "under-developed countr*" or "less-developed countr*" or "third world countr*")  S7 S1 OR S2 OR S3 OR S4 OR S5 OR S6  S6 (MH "Insurance, Health, Reimbursement+")  S5 (MH "Insurance, Health, Reimbursement+")  S4 Health insurance  S3 TI health insurance  S2 (MH “Prospective Payment System+”)  S1 (MM "Insurance, Health+") |
| Language restrictions | None |
| Number of citations | 839 |

| Databases | **Econlit 1886 to September 2015,** |
| --- | --- |
| Host | OvidSP |
| Date of search | 8 September 2016 |
| Years covered | 2010 to September 2016 |
| Search strategy | 1 Health Insurance.kw.  2 health insurance.ti.  3 (social adj5 insurance$).tw.  4 (community adj5 insurance$).tw.  5 (health insurance adj3 program$).tw.  6 universal health insuranc$.tw.  7 affordable health insuranc$.tw.  8 (health insurance adj3 scheme$).tw.  9 micro health insurance$.tw.  10 or/1-9  11 Developing Countries.kw.  12 (developing adj3 (countr$ or nation or nations)).tw.  13 (under-developed adj3 (countr$ or nation or nations)).tw.  14 (less-developed adj3 (countr$ or nation or nations)).tw.  15 (third-world adj3 (countr$ or nation or nations)).tw.  16 low income countr$.tw.  17 middle income countr$.tw.  18 or/11-17  19 (Afghanistan or Guinea-Bisau or Rwanda or Bangladesh or Haiti or Senegal or Benin or Kenya or Sierra Leone or Burkina Faso or Korea or Somalia or Burundi or Kyrgyz Republic or Tajikistan or Cambodia or Lao or Tanzania or Central African Republic or Liberia or Togo or Chad or Madagascar or Uganda or Comoros or Malawi or Uzbekistan or Congo or Mali or Vietnam or Eritrea or Mauritania or Yemen or Ethiopia or Mozambique or Zambia or Gambia or Myanmar or Zimbabwe or Ghana or Nepal or Guinea or Niger).tw.  20 (Albania or Honduras or Paraguay or Angola or India or Philippines or Armenia or Indonesia or Samoa or Azerbaijan or Iran or Sao Tome or Belize or Iraq or Solomon Islands or Bhutan or Jordan or Sri Lanka or Bolivia or Kiribati or Sudan or Cameroon or Kosovo or Swaziland or Cape Verde or Lesotho or Syria$ or China or Maldives or Thailand or Congo or Marshall Islands or Timor-Leste).tw.  21 (Micronesia or Tonga or Djibouti or Moldova or Tunisia or Ecuador or Mongolia or Turkmenistan or Egypt or Morocco or Ukraine or El Salvador or Nicaragua or Vanuatu or Georgia or Nigeria or Gaza or Guatemala or Pakistan or Guyana or Papua New Guinea or "West Bank" or Cote DIvoire).tw.  22 (Algeria or Grenada or Peru or Samoa or Jamaica or Poland or Argentina or Kazakhstan or Romania or Belarus or Latvia or Russian Federation or Bosnia or Herzegovina or Lebanon or Serbia or Botswana or Libya or Seychelles or Brazil or Lithuania or South Africa or Bulgaria or Macedonia or St Kitts or Chile or Malaysia or St Lucia or Colombia or Mauritius or St Vincent or Grenadines or Costa Rica or Mayotte or Suriname or Cuba or Mexico or Turkey or Dominica or Montenegro or Uruguay or Dominican Republic or Namibia or Venezuela or Fiji or Palau or Gabon or Panama).tw.  23 (afghanistan or africa or albania or algeria or angola or antigua or antilles or arab countries or argentina or armenia or asia or asia pacific or azerbaijan or balkans or bangladesh or belarus or belize or benin or bhutan or bolivia or bosnia herzegovina or botswana or brazil or bulgaria or burkina faso or burundi or cambodia or cameroon or caribbean or central africa or central african republic or central america or central asia or chile or china or colombia or congo or costa rica or croatia or cuba).ct.  24 ("democratic republic of the congo" or developing countries or djibouti or dominica or dominican republic or e africa or e asia or e europe or ecuador or egypt or el salvador or eritrea or ethiopia or fiji or gabon or gambia or georgia or ghana or grenada or guatemala or guinea or guinea bissau or guyana or haiti or honduras or india or indonesia or iran or iraq or ivory coast or jamaica or jordan).ct.  25 (kazakhstan or kenya or kiribati or korea or kyrgyzstan or laos or latin america or lebanon or lesotho or liberia or libya or macedonia or madagascar or maghreb or malawi or malaysia or maldives or mali or marshall islands or martinique or mauritania or mauritius or melanesia or mexico or micronesia or middle east or mongolia or morocco or mozambique or myanmar or n africa or n korea or namibia or ne asia or nepal or nicaragua or niger or nigeria or oceania or pacific islands or pakistan or palestine or panama or papua new guinea or paraguay or peru or philippines or polynesia or puerto rico).ct.  26 (syria or tajikistan or tanzania or thailand or timor leste or tobago or togo or tonga or trinidad or "trinidad and tobago" or tunisia or turkey or turkmenistan or uganda or ukraine or uruguay or ussr or uzbekistan or vanuatu or venezuela or vietnam or w africa or w indies or yemen or zambia or zimbabwe).ct.  27 (africa or asia or "latin america and the caribbean").gr.  28 or/19-27  29 18 or 28  30 10 and 29  31 Limit 30 to yr =”2010-Current” |
| Language restrictions | None |
| Number of citations | 486 records |

| Databases | **Embase 1974 to 2016 September,** |
| --- | --- |
| Host | OvidSP |
| Date of search | 8 September 2016 |
| Years covered | 2010 to September 2016 |
| Search strategy | 1 Social Insurance/  2 Public Health Insurance/  3 National Health Insurance/  4 health insurance.ti.  5 (social adj5 insurance$).tw.  6 (community adj5 insurance$).tw.  7 (health insurance adj3 program$).tw.  8 universal health insuranc$.tw.  9 affordable health insuranc$.tw.  10 (health insurance adj3 scheme$).tw.  11 micro health insurance$.tw.  12 or/1-11  13 Developing country/  14 (developing adj3 (countr$ or nation or nations)).tw.  15 (under-developed adj3 (countr$ or nation or nations)).tw.  16 (less-developed adj3 (countr$ or nation or nations)).tw.  17 (third-world adj3 (countr$ or nation or nations)).tw.  18 low income countr$.tw.  19 middle income countr$.tw.  20 or/13-19  20 exp Africa/  21 exp "South and Central America"/  22 Mexico/  23 exp Caribbean Islands/  24 exp Eastern Europe/  25 exp Pacific islands/  26 exp Southeast Asia/  27 exp south asia/  28 middle east/  29 china/  30 korea/  31 mongolia/  32 philippines/  33 taiwan/  34 iran/  35 iraq/  36 jordan/  37 lebanon/  38 palestine/  39 syrian arab republic/  40 "turkey (republic)"/  41 yemen/  42 asia/  43 kazakhstan/  44 kyrgyzstan/  45 tajikistan/  46 turkmenistan/  47 uzbekistan/  48 exp Indian Ocean/  49 (Afghanistan or Guinea-Bisau or Rwanda or Bangladesh or Haiti or Senegal or Benin or Kenya or Sierra Leone or Burkina Faso or Korea or Somalia or Burundi or Kyrgyz Republic or Tajikistan or Cambodia or Lao or Tanzania or Central African Republic or Liberia or Togo or Chad or Madagascar or Uganda or Comoros or Malawi or Uzbekistan or Congo or Mali or Vietnam or Eritrea or Mauritania or Yemen or Ethiopia or Mozambique or Zambia or Gambia or Myanmar or Zimbabwe or Ghana or Nepal or Guinea or Niger).tw.  50 (Albania or Honduras or Paraguay or Angola or India or Philippines or Armenia or Indonesia or Samoa or Azerbaijan or Iran or Sao Tome or Belize or Iraq or Solomon Islands or Bhutan or Jordan or Sri Lanka or Bolivia or Kiribati or Sudan or Cameroon or Kosovo or Swaziland or Cape Verde or Lesotho or Syria$ or China or Maldives or Thailand or Congo or Marshall Islands or Timor-Leste).tw.  51 (Micronesia or Tonga or Djibouti or Moldova or Tunisia or Ecuador or Mongolia or Turkmenistan or Egypt or Morocco or Ukraine or El Salvador or Nicaragua or Vanuatu or Georgia or Nigeria or Gaza or Guatemala or Pakistan or Guyana or Papua New Guinea or "West Bank" or Cote DIvoire).tw.  52 (Algeria or Grenada or Peru or Samoa or Jamaica or Poland or Argentina or Kazakhstan or Romania or Belarus or Latvia or Russian Federation or Bosnia or Herzegovina or Lebanon or Serbia or Botswana or Libya or Seychelles or Brazil or Lithuania or South Africa or Bulgaria or Macedonia or St Kitts or Chile or Malaysia or St Lucia or Colombia or Mauritius or St Vincent or Grenadines or Costa Rica or Mayotte or Suriname or Cuba or Mexico or Turkey or Dominica or Montenegro or Uruguay or Dominican Republic or Namibia or Venezuela or Fiji or Palau or Gabon or Panama).tw.  53 or/12-52  54 or/1-11  55 53 and 54  56 limit 55 to yr="2010 -Current" |
| Language restrictions | None |
| Number of citations | 3913 records |

| Databases | **Ovid MEDLINE(R)** |
| --- | --- |
| Host | OvidSP |
| Date of search | 8 September 2016 |
| Years covered | January 2010 to September 2016 |
| Search strategy | 1 Insurance, Health/  2 Insurance, Health, Reimbursement/  3 Insurance, Hospitalization/  4 Insurance, Major Medical/  5 Insurance, Physician Services/  6 Insurance, Surgical/  7 Single-Payer System/  8 exp Insurance Coverage/  9 health insurance.ti.  10 (social adj5 insurance$).tw.  11 (community adj5 insurance$).tw.  12 (health insurance adj3 program$).tw.  13 universal health insuranc$.tw.  14 affordable health insuranc$.tw.  15 (health insurance adj3 scheme$).tw.  16 micro health insurance$.tw.  17 or/1-16  18 Developing Countries/  19 (developing adj3 (countr$ or nation or nations)).tw.  20 (under-developed adj3 (countr$ or nation or nations)).tw.  21 (less-developed adj3 (countr$ or nation or nations)).tw.  22 (third-world adj3 (countr$ or nation or nations)).tw.  23 low income countr$.tw.  24 middle income countr$.tw.  25 or/18-24  26 exp Africa/  27 exp South America/  28 exp Central America/  29 Mexico/  30 Latin America/  31 exp caribbean region/  32 exp Europe, Eastern/  33 pacific islands/  34 exp melanesia/  35 exp asia, central/  36 exp asia, southeastern/  37 exp asia, western/  38 exp china/  39 mongolia/  40 exp indian ocean islands/  41 or/25-40  42 (Afghanistan or Guinea-Bisau or Rwanda or Bangladesh or Haiti or Senegal or Benin or Kenya or Sierra Leone or Burkina Faso or Korea or Somalia or Burundi or Kyrgyz Republic or Tajikistan or Cambodia or Lao or Tanzania or Central African Republic or Liberia or Togo or Chad or Madagascar or Uganda or Comoros or Malawi or Uzbekistan or Congo or Mali or Vietnam or Eritrea or Mauritania or Yemen or Ethiopia or Mozambique or Zambia or Gambia or Myanmar or Zimbabwe or Ghana or Nepal or Guinea or Niger).tw.  43 (Albania or Honduras or Paraguay or Angola or India or Philippines or Armenia or Indonesia or Samoa or Azerbaijan or Iran or Sao Tome or Belize or Iraq or Solomon Islands or Bhutan or Jordan or Sri Lanka or Bolivia or Kiribati or Sudan or Cameroon or Kosovo or Swaziland or Cape Verde or Lesotho or Syria$ or China or Maldives or Thailand or Congo or Marshall Islands or Timor-Leste).tw.  44 (Micronesia or Tonga or Djibouti or Moldova or Tunisia or Ecuador or Mongolia or Turkmenistan or Egypt or Morocco or Ukraine or El Salvador or Nicaragua or Vanuatu or Georgia or Nigeria or Gaza or Guatemala or Pakistan or Guyana or Papua New Guinea or "West Bank" or Cote DIvoire).tw.  45 (Algeria or Grenada or Peru or Samoa or Jamaica or Poland or Argentina or Kazakhstan or Romania or Belarus or Latvia or Russian Federation or Bosnia or Herzegovina or Lebanon or Serbia or Botswana or Libya or Seychelles or Brazil or Lithuania or South Africa or Bulgaria or Macedonia or St Kitts or Chile or Malaysia or St Lucia or Colombia or Mauritius or St Vincent or Grenadines or Costa Rica or Mayotte or Suriname or Cuba or Mexico or Turkey or Dominica or Montenegro or Uruguay or Dominican Republic or Namibia or Venezuela or Fiji or Palau or Gabon or Panama).tw.  46 42 or 43 or 44 or 45  47 41 or 46  48 17 and 47  49 Limit 48 to yr=”2010-Current” |
| Language restrictions | None |
| Number of citations | 1963 |

| Databases | **Science Citation Index Expanded (SCI-EXPANDED)**  **Social Sciences Citation Index (SSCI)**  **Arts & Humanities Citation Index (A&HCI)**  **Conference Proceedings Citation Index- Science (CPCI-S)**  **Conference Proceedings Citation Index- Social Science & Humanities (CPCI-SSH)** |
| --- | --- |
| Host | ISI Web of Science |
| Date of search | 8 September 2016 |
| Years covered | January 2010 to September 2016 |
| Search strategy | #21 #16 or #17 or #18 or #19 or #20  #20 #15 AND #5  #19 #15 AND #4  #18 #15 AND #3  #17 #15 AND #2  #16 #15 AND #1  #15 #6 or #7 or #8 or #9 or #10 or #11 or #12 or #13 or #14  #14 ts=(public same "health insurance")  #13 ts=(micro* same "health insurance")  #12 ts="affordable health insurance"  #11 ts="universal health insurance"  #10 ts=("health insurance" same program*)  #9 ts=("health insurance" same scheme*)  #8 ti="health insurance"  #7 ts= (Community SAME "health insurance")  #6 ts=(social same "health insurance" )  #5 TS=(Algeria or Grenada or Peru or Samoa or Jamaica or Poland or Argentina or Kazakhstan or Romania or Belarus or Latvia or Russia or Bosnia or Herzegovina or Lebanon or Serbia or Botswana or Libya or Seychelles or Brazil or Lithuania or Africa or Bulgaria or Macedonia or "St Kitts" or Chile or Malaysia or "St Lucia" or Colombia or Mauritius or "St Vincent" or Grenadines or "Costa Rica" or Mayotte or Suriname or Cuba or Mexico or Turkey or Dominica or Montenegro or Uruguay or "Dominican Republic" or Namibia or Venezuela or Fiji or Palau or Gabon or Panama)  #4 TS=(Albania or Honduras or Paraguay or Angola or India or Philippines or Armenia or Indonesia or Samoa or Azerbaijan or Iran or "Sao Tome" or Belize or Iraq or "Solomon Islands" or Bhutan or Jordan or "Sri Lanka" or Bolivia or Kiribati or Sudan or Cameroon or Kosovo or Swaziland or "Cape Verde" or Lesotho or Syria* or China or Maldives or Thailand or Congo or "Marshall Islands" or Timor-Leste)  #3 TS=(Micronesia or Tonga or Djibouti or Moldova or Tunisia or Ecuador or Mongolia or Turkmenistan or Egypt or Morocco or Ukraine or "El Salvador" or Nicaragua or Vanuatu or Georgia or Nigeria or Gaza or Guatemala or Pakistan or Guyana or "Papua New Guinea" or "West Bank" or Cote DIvoire)  #2 TS=(Afghanistan or Guinea-Bisau or Rwanda or Bangladesh or Haiti or Senegal or Benin or Kenya or "Sierra Leone" or "Burkina Faso" or Korea or Somalia or Burundi or Kyrgyz or Tajikistan or Cambodia or Lao or Tanzania or "Central African Republic" or Liberia or Togo or Chad or Madagascar or Uganda or Comoros or Malawi or Uzbekistan or Congo or Mali or Vietnam or Eritrea or Mauritania or Yemen or Ethiopia or Mozambique or Zambia or Gambia or Myanmar or Zimbabwe or Ghana or Nepal or Guinea or Niger)  #1 ts=("developing countr*" or "developing countr*" or "low income countr*" or "middle income countr*" or "under-developed countr*" or "less-developed countr*" or "third world countr*") |
| Language restrictions | None |
| Number of citations | 1313 records |
