# Supplementary material for: The impact of public health insurance on health care utilisation, financial protection and health status in low- and middle-income countries: A systematic review
Source: PLoS One. 2019 Aug 28;14(8):e0219731. doi: 10.1371/journal.pone.0219731 (PMC6713352; doi:10.1371/journal.pone.0219731)
Supplement: S2 Table — (DOCX) [file pone.0219731.s002.docx]

S2 Table. Study Characteristic and Reported Effect from the Included Studies (N = 68)

| **ID** | **Study** | **Type of Study** | **QUEENS** | **GRADE** | **Utilization** | **Financial Protection** | **Health Outcomes** |
| --- | --- | --- | --- | --- | --- | --- | --- |
| 1 | Babiarz et al (2010). Country: China. Scheme: SHI | Difference in difference (DID). Data: Household Survey. N=6,201 | 2 | Low | 5% increase in village clinics use, but no change in overall medical care use. | OOP spending fell by 19%; 2%-point reduction in the likelihood of **catastrophic health expenditure** and 2%-point reduction in the likelihood of financing medical care through asset sales or borrowing |  |
| 2 | Galarraga et al. (2010). Country: Mexico. Scheme: SHI | Instrumental Variable (IV), bivariate probit, and two-stage residual inclusion (2SRI). Data: Household survey. N=20,792 | 2 | Low |  | First database: Outpatients expenses decreased by 447 pesos; hospitalization by 450 pesos; and medicines by 111 pesos per year; Reduced **catastrophic health expenditure** event by 4.6 % points with bivariate probit and 4.7 % points with 2SRI. Second database: Conclusion is similar, but lower magnitude. 171; 175; 360; 3.6; 3.7 respectively |  |
| 3 | Koch and Alaba (2010). Country: South Africa. Scheme: SHI | Propensity Score Matching (PSM). Data: Household survey. N=4,780 | 1 | Low |  | Rural households would accommodate the increased insurance burden by either decreasing other food expenditures by about 20% or by decreasing their transportation and communication budgets by about 15%. Similar result for urban population |  |
| 4 | Trujillo et al. (2010). Country: Colombia. Scheme: SHI | IV. Data: Household survey. N=1,438 | 1 | Low | People in contributory system were 25.9 percent more likely to report use of preventive visits compared with people in subsidized system. People in subsidized system is not likely to take preventive effort compared with uninsured population. Ex ante moral hazard is suggested |  |  |
| 5 | Hou and Chao (2011) Country: Georgia. Scheme: SHI | Regression Discontinuity (RD) and two-part model (2PM) Data: Administrative data N: 29,460 | 3 | Medium | The beneficiaries are nine time more likely to utilize acute surgeries compared to non-beneficiaries. The poorest are 47% more likely to have acute surgery compared to the richest and 13% more likely to have planned surgeries. No significant difference for baby deliveries |  |  |
| 6 | Panpiemras et al. (2011). Country: Thailand. Scheme: SHI | Fixed effect. Data: Household survey. N=63,360 | 1 | Low | The number of outpatients increased by 55.98% while the outpatient visits increased by 41.34% after the scheme was implemented. However, similar results were not found for inpatients. The effect of the scheme faded away quickly in the subsequent years. |  |  |
| 7 | Quimbo et al. (2011) Country: The Philippines Scheme: SHI | DID. Data: Randomized study. N = 1100 | 3 | Medium |  |  | Being confined in an intervention hospital decreases the likelihood of having positive C-Reactive protein and the likelihood of wasting (low weight-for-height ratio) by 4 and 9 percentage points respectively |
| 8 | Sepehri et al. (2011). Country: Vietnam. Scheme: CBHI | Panel random effect. Data: Household survey. N=6,037 | 1 | Low |  | In fixed effect model, contributory and voluntary groups did not have any impact on OOP, but HIP (i.e. poor people entitled to free health care) have 16% reduction effects |  |
| 9 | Sosa-Rubi, Salinas-Rodriguez, and Galarraga. (2011). Country: Mexico. Scheme: SHI | DID and IV. Data: Household. N= ? | 2 | Low |  | At the local level the authors did not find any effect of the scheme. At the household level, they found a protective effect of the scheme on catastrophic health expenditure and the out-of-pocket health payments in outpatient and hospitalization in rural areas; and a significant effect on the reduction of OOP payments in outpatient health services in urban areas. |  |
| 10 | Blanchet et al. (2012). Country: Ghana. Scheme: SHI | PSM. Data: Household survey. N=2543 | 1 | Low | Keeping all other factors constant, the enrolled women are 40 % more likely to have attended a clinic over the past year and they have about 57 % more prescriptions. Most remarkably, the enrolled women appear nearly twice as likely (83 per cent more likely) to have stayed overnight at a hospital than non-enrolled women |  |  |
| 11 | Chen and Jin (2012). Country: China. Scheme: SHI | DID-PSM. Data: Census. N=3977 - 950,681 | 3 | Low |  |  | The new scheme (NCMS) has no obvious effect in reducing the incidence of child or maternal mortality at the village level. The NCMS has no significant effect on school enrolment once we control for the endogenous introduction and take-up of the NCMS |
| 12 | Dhillon et al. (2012). Country: Rwanda. Scheme: CBHI | FD with IV (Arellano-Bond GMM). Data: Household survey. N=84-950 | 1 | low | The removal of financial barriers had the single greatest impact on monthly utilisation rates. The effect of the event of removing financial barriers resulted in a near 100% increase in utilisation compared to before the intervention |  |  |
| 13 | Fan et al. (2012) Country: India Scheme: SHI | DID, Quintile Regression, and matching method. Data: Household Survey. N= 11,881 | 3 | Low |  | Households in the first intervention area experienced significantly reduced per capita per month inpatient health expenditure by Rs. 12, but not outpatient spending. The first 2 months of implementation of the second intervention did not significantly reduce per capita inpatient spending (with effects in the expected direction), although it significantly reduced per capita outpatient drug spending. The probability of having an inpatient health expenditure account for 50 % or more of total health expenditure and having total health expenditure exceeding 15 % or more of total household spending was reduced in the first intervention area. No effects are detected on our measure of impoverishment in any panel. |  |
| 14 | Lu et al. (2012). Country: Rwanda. Scheme: CBHI | PSM; 2SRI; IV. Data: Household survey. N=1,000 - 6,334 | 2 | Low | The odds of using medical care increased by 2 for the enrolees. Among under-five children that reported diarrhoea, fever, or acute respiratory infection in the two weeks prior to the survey, the enrolees were more likely to use medical care. Among women who delivered children in the survey years, the enrolees were more likely to use skilled-birth attendance. | The enrolled households were less likely to incur catastrophic health spending. |  |
| 15 | Lu, Liu, and Shen (2012). Country: China. Scheme: SHI | Two-part model and hurdle model with PSM and IV. Data: Household survey. N=1170 - 2740 | 2 | Low | We have found that the new scheme has had a significant and positive effect on the probability of seeking medical care and the number of visits with its marginal effect equals to 1.20 | The new scheme did not seem to have had a significant impact on households’ out-of-pocket health expenditure and on reducing catastrophic spending |  |
| 16 | Nguyen (2012). Country: Vietnam. Scheme: CBHI | PSM combined with DID; Compared with IV and Fixed effect separately. Data: Household survey. N=6780 | 3 | Low | The voluntary health insurance helps the insured people increase their annual outpatient and inpatient visits by around 45% and 70%, respectively. | The effect of voluntary health insurance on out‐of‐pocket expenses on health care services is not statistically significant |  |
| 17 | Parmar et al. (2012). Country: Burkina Faso. Scheme: CBHI | Fixed effect and IV. Data: Household survey. N=890 | 2 | Low |  | In the 2SLS, insurance had a positive effect on per capita household assets (24.6 percent). By FE, insurance increased per capita household assets by 1 percent at 10 percent significant level |  |
| 18 | Robyn et al. (2012). Country: Burkina Faso. Scheme: CBHI | Fixed effect. Data: Household survey. N=2820 | 3 | Medium | Individuals residing in areas with insurance scheme were marginally more likely to seek treatment in general and to seek facility-based professional care but both effects are not statistically significant at 5% level. |  |  |
| 19 | Robyn et al. (2012). Country: Burkina Faso. Scheme: CBHI | Multinomial Logit and PSM. Data: Household survey. N=1240 | 1 | Low | While enrolment may lead to improved access to facility care within the formal health system, the insured population continues to actively seek drugs from the informal sector, resulting in a continued high prevalence of self-medication within the household. |  |  |
| 20 | Wirtz et al. (2012). Country: Mexico. Scheme: SHI | Heckman selection model and PSM&IV. Data: Household survey. N=28,260 | 2 | Low |  | Being affiliated with the scheme reduced the probability of incurring catastrophic health expenditure by 9.7% and the annual amount spent for medicine by US$24.51 but showed no significant effect on the percentage of health expenditure on the acute emergencies in comparison to households without health insurance. |  |
| 21 | Yilma et al. (2015). Country: Ethiphia. Scheme: CBHI | Fixed effect and PSM. Data: Household survey. N=9455 | 1 | Low |  | The authors found a reduced probability of having outstanding loans ranging between 4% and 5%, depending on methods and control groups, which translates to about 13% of baseline values. There are also negative coefficients for the amount of outstanding loans although these are imprecise. Estimates for all types of livestock are not statistically significant. |  |
| 22 | Aji et al. (2013). Country: Indonesia. Scheme: SHI | IV and Fixed effect. Data: Household survey. N=6335 | 2 | Low |  | The health insurance for poor people decreased out-of-pocket expenditures by 34% compared to the uninsured |  |
| 23 | Avilla-Burgos et al. (2013). Country: Mexico. Scheme: SHI | PSM. Data: Household survey. N=12,250 | 1 | Low |  | The insurance scheme reduces the likelihood of health expenditure in 3.6 and 7.1% in households with patients diagnosed with DM and/or hypertension, respectively. |  |
| 24 | Camacho and conover (2013). Country: Colombia. Scheme: SHI | RD. Data: Household survey. N=40,931 | 3 | Low |  |  | The subsidised scheme had a significant and positive effect on health, reducing the incidence of low birth weight between 1.7 and 3.8 percentage points. They also found that the direction of the impact on very low birth weight, an indicator for the baby being preterm, and for 5-minute Apgar score show improved newborn health, but these results are not always significant |
| 25 | Cheung and Padieu (2013). Country: China. Scheme: SHI | OLS and IV. Data: Household survey. N=933 | 2 | Low |  | They found a significant and negative impact of insurance on participant's saving in third quantiles. No significant impact on the poorest nor the richest. No significant effect on health expenditure regardless of its income quantiles |  |
| 26 | Fink et al. (2013). Country: Burkina Faso. Scheme: CBHI | IV. Data: Randomized study. N=12,118 | 3 | Medium |  | The insurance rollout was associated with about a 30% reduction in the likelihood of catastrophic expenditure. | The introduction of the insurance scheme did not have any effect on health outcomes for children and young adults, but appears to have increased mortality among individuals aged 65 and older. |
| 27 | Hassan et al. (2013). Country: Colombia. Scheme: SHI | Poisson and probit with two-stage nonlinear method of moments. Data: Household survey. N=12,975 | 1 | Low | The insured group visited a doctor 21% less frequently for preventive health care purposes than the uninsured group. Also, there is a positive effect, which means that individuals enrolled in this program have a higher probability of hospitalization. |  |  |
| 28 | Jing et al. (2013). Country: China. Scheme: SHI | DID. Data: Household survey. N=1681 | 1 | Low |  | The reimbursement policies for chronic disease in the health insurance programs were not significantly effective in reducing household catastrophic health expenditure event. |  |
| 29 | Miller et al. (2013). Country: Colombia. Scheme: SHI | Fuzzy RD and IV. Data: Household survey. N=4219 and 3334 | 3 | Low | The insured is associated with a 29 percentage point increase in the probability of a preventive physician visit in the past year, a 13 percentage point increase in reported physician visits because of health problems within the past 30 days, and a 1.50 more growth-monitoring and well-care visits in the past year compared to uninsured children | The insured lowered mean inpatient spending by about 60,000 pesos (α=0.10) or 30 % reduction. There is no statistically significant association for outpatient care. | The insured is associated with 1.4 fewer child days absent from usual activities due to illness in the past month (α=0.05). Enrolment is also associated with an 18 percentage point reduction in the self-reported incidence of cough, fever, or diarrhoea among children in the preceding two weeks (but it is not statistically significant at 5% levels |
| 30 | Nguyen and Wang (2013). Country: Vietnam. Scheme: SHI | DID. Data: Household Survey. N=628 - 1209 | 2 | Low | The use of secondary hospitals increased significantly for both 0–3 and 4–5 years age groups by 0.02 and 0.03 admissions per year, respectively. Number of sick days among the age group 4–5 years reduced by 0.81 because of the health insurance scheme. Compared with the baseline value of 3.06, this represents 26% reduction. | The probability of having large OOP in the insured group went down by 1.7 percentage point, which represents nearly 60% of the baseline value (0.027). |  |
| 31 | Sparrow et al. (2013). Country: Indonesia. Scheme: SHI | DID and PSM. Data: Household survey. N=34,525 | 2 | Low | The health insurance scheme increased outpatient utilization by 0.062 visits per person per month (vppm) while kernel matching yields an impact of 0.079 vppm. | The scheme seems to increase OOP payments and budget shares, particularly in urban areas, although the propensity score matching results show larger standard errors. Evidence of increased incidence of catastrophic spending at a 15 percent threshold, but not significant with PSM |  |
| 32 | Zoidze et al. (2013). Country: Georgia. Scheme: SHI | DID. Data: household survey. N=? | 1 | Low | Individuals with acute sicknesses in the preceding 30 days are more likely to report using health services and report a substantial increase in utilisation from 2007, with 66% consulting a health care provider in 2010 compared with 57% in 2007. However, the individuals with chronic illnesses utilised less services resulting in zero net effect for an overall use of services | Less OOP expenditure for inpatient services (−227 Gel per episode) and total monthly health care payments (−27 Gel), but a dramatic increase in the shares of the general population facing the catastrophic health expenditure from 11.7% in 2007 to 24.8% in 2010 |  |
| 33 | Abrokwah et al. (2014). Country: Ghana. Scheme: SHI | OLS with proxy and TPM. Data: Household survey. N=1012 | 1 | Low | Having insurance increases the number of prenatal care visits by 24% relative to being uninsured. | The predicted total out-of-pocket spending on prenatal care for an insured pregnant woman is about 3,600 cedis ($0.40) for her first prenatal care visit, while the predicted total spending for an uninsured pregnant woman stands at about 21,600 cedis ($2.40) for her first visit |  |
| 34 | Bai and Wu (2014). Country: China. Scheme: SHI | DID and matching method. Data: Household survey. N=17,715 | 3 | Low |  | Insurance coverage on average stimulated nonmedical consumption by 5.5% for the insured (p<0.1). The insurance effect does not appear to be driven by the crowd-in story because the insurance effect is substantial even for households that do not incur medical expenditures. The insurance effect increases with the generosity of insurance coverage at the county hospitals. |  |
| 35 | Bernal et al., (2014). Country: Peru. Scheme: SHI | Fuzzy RD. Data: Household survey. N=4161 | 3 | Low | Being covered by health insurance increases the probability of visiting a doctor in the four weeks prior to the interview by 51.5 percentage points. The probability of being vaccinated in the three months prior to the interview increases by 28.9 percentage points, and women at fertile age are 65.0 percentage points more likely to control their pregnancy in the previous twelve months | We find that health insurance coverage increases the probability that individual health expenditures exceed 5 and 10 percent of the per capita household income by 40.6% and 29.1% respectively. Overall, the authors never find a negative effect on either expected health expenditures or measures of variability or risk of high expenditures. |  |
| 36 | Chen et al. (2014). Country: China. Scheme: SHI | Bivariate probit. Data: Household survey. N=43,381 | 2 | Low | Being insured increased the likelihood of hospitalisation in the past year (1.8% probit), increased the likelihood of refused inpatient treatment in the past year, and increased outpatient health services utilisation (1.7%). |  |  |
| 37 | Guindon (2014). Country: Vietnam. Scheme: SHI | DID, FE, and DID-PSM; TPM. Data: Household survey. N=6775 | 2 | Low | Overall, the insurance scheme is not found to have a significant effect on the utilization of outpatient and inpatient services. Using PSM the magnitude is smaller for outpatient care, but bigger for inpatient admissions. |  |  |
| 38 | Hendriks et al. (2014). Country: Nigeria. Scheme: CBHI | DID. Data: Household survey. N=413 | 1 | Low |  |  | Systolic blood pressure decreased by 10.41 mm Hg (P < .001) from 2009 to 2011 in the program area. This reduction was 5.24 mm Hg (P = .02) greater compared with the control area. Diastolic blood pressure decreased by 4.27 mm Hg (P < .001) in the program area, 2.16mmHg ( P = .04) greater reduction compared with the reduction in the control area, where diastolic blood pressure decreased by 2.11 mm Hg. |
| 39 | Hou et al. (2014). Country: China. Scheme: SHI | Fixed effect with Two part model and Generalized Linear Model. Data: Household survey. N=1,478 - 28,824 | 2 | Low | A 1-percentage point increase in insurance generosity raises the probability of using inpatient care by 0.004, which corresponds to an increase of about 7.5% compared with the overall sample probability (0.053). The social pooling account (SPA) system for outpatient care proves to be more effective in improving access than the system of household medical saving account (MSA). | No effects are found of health insurance on spending in the full sample, but conditional upon use, NCMS reduces the share of OOP spending for an outpatient visit. More worrisome is that we find higher NCMS cover to raise total spending per hospitalization and to increase the OOP spending per inpatient stay (among the users) |  |
| 40 | Liu and Zhao (2014). Country: China. Scheme: SHI | IV. Data: Household survey. N=628 | 2 | low | Participation in the health insurance scheme has significantly increased the probability of individuals’ use of formal medical care, by 10–15 percentage points, increased likelihood of outpatient by 7-13%, and increased the number of inpatient days by 0.35-0.5 days. | Joining the health insurance scheme resulted in an increase in out-of-pocket health expenditures of about 11–172 RMB, although the coefficients are insignificant at 5% level. |  |
| 41 | Liu, Wu, and Liu (2014). Country: China. Scheme: SHI | Structural equation modelling. Data: survey. N-1645 | 1 | low |  | Taking the direct and indirect effects together, patients enrolled in the scheme had to pay 351 yuan more out-of-pocket than the uninsured, although this total effect was statistically insignificant |  |
| 42 | Nguyen (2014). Country: Vietnam. Scheme: SHI | PSM. Data: Household survey. N=15,550 | 1 | Low | On average, a person with compulsory insurance pays 0.47 visits to health care facilities more than their uninsured counterpart. There is no moral hazard or adverse selection in using inpatient services. |  |  |
| 43 | Pfutze (2014). Country: Mexico. Scheme: SHI | Weighted Exogenous Sampling Max Likelihood. Data: Household survey. N=836,809 | 2 | Low |  |  | The risk of a child dying in the first month of life is reduced by close to 5 out of 1,000 (or 0.5%) for the population at large and by around 7 out of 1,000 (0.7%) for the program’s target population. |
| 44 | Sheth. (2014) Country: India. Scheme: CBHI | DID. Data: Randomised Trial. N=1311 | 3 | Low | The author found limited suggestive evidence of the insurance possibly reducing the consumption of health care. He also found no statistically significant evidence that the effects of the insurance offer are increasing over time. | He found limited suggestive evidence of the insurance possibly reducing the expenditure of health care. |  |
| 45 | Sood et al. (2014). Country: India. Scheme: SHI | RD and DID. Data: Household survey. N=986 - 22,796 | 2 | low | The point estimate was large which could suggest of a positive effect on utilization, but it is not significant at 5% level. | The scheme was associated with a 34% reduction in out-of-pocket health expenditure for admission to hospital for covered conditions. | The mortality rate from conditions covered by the scheme was 0.32% in eligible households compared with 0.90% in ineligible households (difference of 0.58 percentage points, 95% confidence interval 0.40 to 0.75; P<0.001; 64% risk reduction). |
| 46 | Yuan et al. (2014). Country: China. Scheme: SHI | DID. Data: Adminstrative data. N=720 | 2 | Low |  | The DID regression analysis showed no impact of benefit design on patients’ out-of-pocket expenses, which indicates that the expansion of public health insurance coverage did not have any effect on relieving financial burden of disease for patients. |  |
| 47 | Alkenrack and Lindelow (2015). Country: Lao. Scheme: CBHI | PSM. Data: Household survey. N=3000 | 2 | Low | The insured was almost twice as likely as the uninsured to have had an inpatient visit in a 1-year period. Regarding source of care, the matched estimates show that the insured was significantly more likely than the uninsured to visit both district and provincial hospitals, for both inpatient and outpatient care. | The author found no significant difference in expenditures between the insured and uninsured. This lack of difference in spending indicates positive financial protection in that the insured are using more services, paying less at the point of service, but spending approximately the same amount overall as the uninsured. |  |
| 48 | Atella, Brugiavini, and Pace. (2015). Country: China. Scheme: SHI | Finite Mixture Model (Bayesian). Data: Household survey. N=3166 | 2 | Low |  | The authors found that out-of-pocket expenses decrease only for high income individuals with good health status and the saving rate increases only for low income individuals with good health status. |  |
| 49 | Brugiavini and Pace (2015). Country: Ghana. Scheme: NHI | IV. Data: Household survey. N=9,396 | 2 | Low | The health insurance enrolment positively affects the probability of formal antenatal check-ups before delivery, the probability of delivery in an institution and the probability of being assisted during delivery by a trained person. | On the contrary, the authors found that the health insurance enrolment does not have a significant effect on out-of-pocket expenditure at the extensive margin |  |
| 50 | Cheng et al. (2015). Country: China. Scheme: SHI | PSM and DID. Data: Household survey. N=6598 | 3 | Low | The authors found that the elderly participants are more likely to get adequate medical services when sick, which provides a good explanation for the beneficial health effects of the scheme. | They found no evidence that the scheme has reduced the elderly enrolees’ out-of-pocket spending. | They found no significant effect of the scheme on 3-year mortality for the previously uninsured elderly in scheme-exposed counties, although there is moderate evidence that scheme is associated with reduced mortality for the elderly enrolees. |
| 51 | Fenny et al. (2015). Country: Ghana. Scheme: SHI | Multinomial Logit. Data: household survey. N=11,089 | 1 | Low | The insured are 6 times more likely to choose regional/district hospitals and health centres/clinics and 11 times more likely to choose private hospitals/clinics over informal care when compared to those uninsured and this is significant at 1% level. |  |  |
| 52 | Ghislandi, Manachotphong, and Perego. (2015). Country: Thailand. Scheme: SHI | Triple DD with PS; Double Robust. Data: Household data. N=15,022 | 2 | Low | The insurance increases individuals’ likelihood of having an annual check-up, especially among women. Regarding health care consumption, the insurance increases hospital admissions by over 2% and increases outpatient visits by 13%. |  |  |
| 53 | Gotsadze et al. (2015). Country: Georgia. Scheme: SHI | DID. Data: Household survey. N=11,663 | 1 | Low | The insured was marginally (by 2%) more likely to utilize any formal health care services, but it is not significant at 5% level. | The largest reduction was observed for inpatient spending—at 227 Gel per case. Although most financial impact indicators for the overall sample revealed marginal statistical significance (P value < 0.1) |  |
| 54 | Grogger et al. (2015). Country: Mexico. Scheme: SHI | Logistic regression. Data: Randomised trial. N=83,000 | 3 | Medium |  | In rural areas, remote from health-care facilities, or proximate only to facilities with limited staffing, the programme has not reduced catastrophic out-of-pocket health expenditures. In rural areas proximate to larger facilities, in contrast, the programme has provided considerable financial protection. Also in urban areas, the programme has substantially reduced catastrophic out-of-pocket health spending among beneficiaries |  |
| 55 | Jung and Streeter (2015). Country: China. Scheme: SHI | SSM + 2PM and IV. Data: Household survey. N=50,591 | 2 | Low |  | Our results from a selection model with instrumental variables suggest that having health insurance reduces the expected OOP health expenditure of an individual by 29.42% unconditionally. Meanwhile, conditional on being subjected to positive health expenditure, health insurance helps reduce OOP spending by 44.38%. |  |
| 56 | Limwattananon et al. (2015). Country: Thailand. Scheme: SHI | DID and GLM; censored quintile regression. Data: Household survey. N=26,557 | 2 | Low | They found increase probability of outpatient utilisation among the treatment group. It is almost three percentage points for the elderly, while the point estimate is only half a point and not significant for children. | The reform reduced OOP spending by an average of almost 19 Baht (~$0.47) per person per month and by 28% relative to what spending would have been in the absence of the policy. The reform is estimated to have reduced the probability of spending at least 10% of the household budget on health care by a significant 1.6 percentage points (38%). |  |
| 57 | Makhloufi et al. (2015). Country: Tunisia. Scheme: SHI | PSM. Data: Household survey. N=6538 | 1 | Low | Given illness amongst the enrolees, they found significant increases in outpatient and inpatient care range from 18.8 to 21.4 percentage point and from 25.8 to 38 percentage point, respectively, compared to the uninsured. | The uninsured households appeared to spend almost twice as much on healthcare as the beneficiaries (25.1 for the uninsured vs. 13.85 for the beneficiaries). |  |
| 58 | Palmer et al. (2015). Country: Vietnam. Scheme: NHI | RD. Data: Household survey. N=18,517 | 3 | Low | Insurance increases the probability of an inpatient visit by 6.8% and an outpatient visit by 21.7%; the average number of inpatient and outpatient visits increases by 1.13 and 0.75, respectively. | The impact of insurance on expenditures is positive for both service types. However, the impact is not statistically significant. |  |
| 59 | Pfutze (2014). Country: Mexico. Scheme: SHI | Weighted Exogenous Sampling Max Likelihood. Data: Household survey. N=27,455 | 1 | Low |  |  | For the target population, a one percentage point increase in eligibility is found to decrease miscarriages by .04 percentage point at the average |
| 60 | Yang and Wu (2015). Country: China. Scheme: SHI | Two-part model; PSM and DID; Heckman Selection model. Data: Household survey. N=4084 | 2 | Low |  | The results show a trend of increase in pre-reimbursement outpatient costs (gross billed) between the treatment group compared to the control group (P value < 0.1). The results also show that there is no significant difference in post-reimbursement outpatient costs for the control group and treatment group after insurance reimbursement |  |
| 61 | Yilma et al. (2015). Country: Ghana Scheme: SHI | PSM and Fixed Effect. Data: Household survey. N=761 | 3 | Low | Like the non-parametric estimation, the fixed effects model indicates that in 100 insured households, around 20 people do not sleep under STNs due to insurance uptake. Health insurance apparently increases the benefit of curative care relative to preventive care, and most strongly so if the level of effort, cost and discomfort involved in prevention is higher (the case of STNs). |  |  |
| 62 | Aryeetey et al. (2016). Country: Ghana. Scheme: NHI | IV-FE. Data: Household survey. N=3128 | 2 | Low |  | Our results revealed that enrolment into health insurance reduces household OOPE by 86 %. The effect of health insurance is protective i.e. insured households were 3 % less likely to incur catastrophic expenditure and 7.5 % less likely to fall into poverty. |  |
| 63 | Levine, Polimeni, and Ramage (2016). Country: Cambodia. Scheme: CBHI | IV. Data: Randomized study. N=5,000 | 3 | Medium | Insured households were 15.8 percentage points more likely to use a health centre for first treatment (P < 0.001) and 10.7 and 8.0 percentage points less likely to visit a private doctor or drug seller for first treatment compared to the control group. No statistically significant impact on first treatment at a public hospital. | No impact on overall wealth; households that purchased insurance due to the steep discount were 10.8 percentage points less likely than the control to have at least one of these adverse economic outcomes | There is no significant difference between treated and control households for this index of health measures |
| 64 | Liao, Gilmour, and Shibuya (2015). Country: China. Scheme: SHI | PSM. Data: Household Survey. N=9971 | 1 | Low | Among diagnosed hypertensives, health insurance increased the probability that they would receive treatment by 28.7% (95% CI: 10.6–46.7%, p-value 0.001) compared to the uninsured. |  |  |
| 65 | Nguyen (2016). Country: Vietnam. Scheme: NHI | FE. Data: Household survey. N=5013 | 2 | Low | In 2010-2012, the student health insurance and free health insurance programs increased the number of health care visits of children by approximately 13.6 and 66.1 %, respectively. | Having free health insurance reduced the out-of-pocket health expenditures per visit by around 15.8 in the period 2006–2008 and 63.4 % in the period 2010–2012. Student insurance has no effect. |  |
| 66 | Peng and Conley (2015). Country: China. Scheme: SHI | IV and DID. DataL Household survey. N=8309 | 2 | Low |  |  | The scheme significantly decreased children’s malnutrition probability by 6.5 % points.  Our DID estimation shows that the average 3-day food consumption for women at child-bearing age who live in the counties that enrolled in the NCMS in 2006 increased by 180 calories, while the effect is not significant for those counties that rolled out the program in 2004. |
| 67 | Raza et al. (2016). Country: India. Scheme: CBHI | IV. Data: Randomized study. N=21,372 | 3 | Medium | The schemes had no impact on access to outpatient or inpatient care. | They do not find any significant impact on healthcare expenditure. |  |
| 68 | Rivera-Hernandez et al. (2016) Country: Mexico. Scheme: SHI | FE-IV. Data: Household survey. N=5307 | 2 | Low | The effect of the scheme on use of insulin and/or oral agents was marginally significant (p = .051), showing a tendency that the insured with diabetes were more likely to use pharmacological treatment. No significant difference was found for antihypertensive medication for the insured as opposed to the uninsured. |  |  |
